# Network analysis: a brief overview and tutorial

**DOI:** 10.1080/21642850.2018.1521283

**Published:** 2018-09-25

**Authors:** David Hevey

**Affiliations:** School of Psychology, Trinity College Dublin, Dublin, Ireland

**Keywords:** Network analysis, *R*, psychological networks, theory of planned behaviour

## Abstract

**Objective***:* The present paper presents a brief overview on network analysis as a statistical approach for health psychology researchers. Networks comprise graphical representations of the relationships (edges) between variables (nodes). Network analysis provides the capacity to estimate complex patterns of relationships and the network structure can be analysed to reveal core features of the network. This paper provides an overview of networks, how they can be visualised and analysed, and presents a simple example of how to conduct network analysis in *R* using data on the Theory Planned Behaviour (TPB).

**Method**: Participants (*n* = 200) completed a TPB survey on regular exercise. The survey comprised items on attitudes, normative beliefs, perceived behavioural control, and intentions. Data were analysed to examine the network structure of the variables. The EBICglasso was applied to the partial correlation matrix.

**Results**: The network structure reveals the variation in relationships between the items. The network split into three distinct communities of items. The affective attitude item was the central node in the network. However, replication of the network in larger samples to produce more stable and robust estimates of network indices is required.

**Conclusions**: The reported network reveals that the affective attitudinal variable was the most important node in the network and therefore interventions could prioritise targeting changing the emotional responses to exercise. Network analysis offers the potential for insight into structural relations among core psychological processes to inform the health psychology science and practice.

## Introduction

Health psychology research examines how the complex interactions between biological, psychological, and social factors influence health and well-being. For example, the UK Foresight map of obesity (see https://www.gov.uk/government/collections/tackling-obesities-future-choices) provides a comprehensive representation of the complex system of over 300 relationships between over 100 variables and obesity (Finegood, Merth, & Rutter, 2010). The developers of the map assumed that obesity is the result of the interplay between a wide variety of factors, including a person’s physical make-up, eating behaviour, and physical activity pattern. The system reflects the relevant factors and their interdependencies that produce obesity as a behavioural outcome. The variables were classified into various categories of causal factors; for example, social psychological factors (e.g. peer pressure), individual psychological factors (e.g. stress), environmental factors (e.g. the extent to which one’s environment makes it easy to engage in regular walking), and individual physical activity factors (e.g. functional fitness). On the basis of expert academic opinion the Foresight report authors proposed that the variables in the system not only influence obesity, but can also have positive (e.g. high levels of stress cause high levels of alcohol consumption) and negative (e.g. high levels of stress cause low levels of physical activity) effects on each other, some have distal effects whereas others have proximal effects, and effects can be unidirectional (e.g. social attitudes towards fatness causes conceptualisations of obesity as an illness) or reciprocal (e.g. physical activity causes functional fitness, which causes physical activity). Networks are a fundamental characteristic of such complex systems; consequently, health psychological science can benefit from considering the network structure of the phenomena that it seeks to understand. It has been argued that networks pervade all aspects of human psychology (Borgatti, Mehra, Brass, & Labianca, 2009), and in the past decade network analysis has become an important conceptual and analytical approach in psychological research. Although network analysis has a long history of being applied in causal attribution research (e.g. Kelly, 1983) and social network analysis (Clifton & Webster, 2017), its broader potential for psychological science was highlighted over a decade ago by van der Maas et al. (2006). The frequently reported patterns of positive correlations between various cognitive tasks (e.g. verbal comprehension and working memory) are typically explained in terms of a dominant latent factor, i.e. the correlations reflect a hypothesised common factor of general intelligence (*g*). However, van der Maas and colleagues argued that this empirical pattern can also be accounted for by means of a network approach, wherein the patterns of positive relationships can be explained using a mutualism model, i.e. the variables have mutual, reinforcing, relationships. From a network analysis perspective, the network of relationships between the variables constitute the psychological phenomenon (De Schryver, Vindevogel, Rasmussen, & Cramer, 2015), which is a *system* wherein the constituent variables mutually influence each other without the need to hypothesise the existence of causal latent variables (Schmittmann et al., 2013). In addition to addressing psychometric issues (Epskamp, Maris, Waldorp, & Borsboom, In Press) network perspectives can inform other areas of psychological science.

A key impetus for the current research on networks in psychology derives from Borsboom and colleagues’ influential application of networks in the field of clinical psychology in relation to psychopathology symptoms (e.g. Borsboom, 2017; Borsboom & Cramer, 2013; Cramer et al., 2016; Cramer, Waldorp, van der Maas, & Borsboom, 2010). Network models are also increasingly applied in other areas such as health related quality of life (HRQOL) assessment in health psychology (e.g. Kossakowski et al., 2016), personality (e.g. Costantini et al., 2015; Mõttus & Allerhand, 2017), and attitudes (e.g. Dalege et al., 2015). The psychosystems research team (i.e. Denny Borsboom, Angélique Cramer, Sacha Epskamp, Eiko Fried, Don Robinaugh, Claudia van Borkulo, Lourens Waldorp, Han van der Maas) are critical innovators for network analysis in psychology and this paper draws extensively from the key papers from the team and their collaborators; the *psychosystems.org* webpage is an essential resource for anyone interested in network analysis theory, process and applications.

To date, network analysis has not been widely applied in health psychology; however, network models are particularly salient for health psychology because many of the psychological phenomena we seek to understand are theorised to depend upon a large number of variables and interactions between them. The biopsychosocial model (e.g. Engel, 1980) has underpinned health psychology research and theory for the past 4 decades, and it reflects a complex system of mutually interacting and dynamic biological, psychological, interpersonal, and contextual effects on health (Lehman, David, & Gruber, 2017; Suls & Rothman, 2004). From a network perspective, health behaviours and outcomes can be conceptualised as emergent phenomena from a system of reciprocal interactions: network analysis offers a powerful methodological approach to investigate the complex patterns of such relationships. The overall global structural organisation, or topology, of the phenomenon and the roles played by specific variables in the network can be analysed in a manner that other statistical approaches cannot provide. In general, health psychology research, like many areas of psychology, has studied aspects of systems in isolation: for example, using regression models to examine the relationship between focal beliefs and moods and a specific outcome such as health behaviours or adaptation to illness. Although such research provides important insights, this approach is not suited for examining complex systems of interconnected variables and it does not help us easily piece back the various separate research findings on discrete components/sub-pathways into the more complex and complete system. As noted above, the complex interplay of physiological, psychological, social and environmental factors have been highlighted in the context of obesity. Comparable exercises for other chronic illnesses will produce similarly complex networks of variables. Network analysis provides a means to understand system-level relationships in a manner that can enhance psychological science and practice.

Health psychology research often focuses on HRQOL as a key outcome variable and HRQOL is frequently understood as being the common *effect* of observed items in scales, e.g. increased daily pain causes lower mental health. Network analysis has been applied to the SF-36 (Ware & Sherbourne, 1992), a widely used HRQOL scale, to examine the patterns of relationships between the items: Kossakowski et al. (2016) found that the observed covariances between the items may result largely from direct interactions between items. From this perspective, HRQoL emerges from a network of mutually interacting characteristics; the specific nature of the interacting relationships (e.g. causal effect, bidirectional effect, or effects of unmodelled latent variables) requires additional clarification. In addition to offering novel insights into psychometrics, a network approach can be applied to other important health psychology variables (e.g. illness representations, coping strategies) to better understand the nature of the relationships between items used in measurement.

Borsboom’s research on the networks of patterns of interconnected relationships between symptoms of various psychiatric disorders has resulted in the development of a novel network theory of mental disorders (Borsboom, 2017). This theory provides new insights into how trigger events can activate pathways in strongly connected networks to produce symptoms that can become self-sustaining, i.e. because the symptoms are strongly connected, feedback relations between them mean that they can activate each other after the triggering event has been removed. The absence of the trigger may be not be sufficient to de-activate the symptom network and return the person to a state of health; such insights from a network theory of psychopathology can help inform not only understandings of how and why symptoms are maintained, but also how such networks can be targeted to help transition the network back into a healthy state. Of note, such an approach may be beneficial for health psychology approaches to understanding clusters of symptom presentations over time in conditions such as chronic pain and chronic fatigue syndrome.

The network structures of individuals can be visualised and analysed; consequently we may be able to see how the system of beliefs, emotional states, behaviours and symptoms influence each other over time. Systems might comprise sets of variables that are diverse and only marginally connected, or could consist of variables that are highly interconnected. Understanding an individual’s personalised network may allow insight into when an individual’s specific patterns of beliefs and behaviours reach a tipping point, which then negatively impact on mood and symptoms. Such system transitions (e.g. moving from a state of wellness to being impaired functionally) occur gradually in response to changing conditions or they may be triggered by an external perturbation, e.g. life stressor. An individual may have a very robust network so that it remains stable despite the perturbations (e.g. symptom flare up) and consequently the person can maintain function, whereas other individuals may have less resilient networks wherein it is challenging to restore disturbed equilibrium. How such networks evolve over time and respond to changes in key and peripheral variables cannot be understood using traditional analytical methods: network analysis offers rich potential to further our understanding of complex systems of relationships among variables.

The Causal Attitude Network (CAN) model, which conceptualises attitudes as networks of causally interacting evaluative reactions (i.e. beliefs, feelings, and behaviours towards an attitude object; Dalege et al., 2015), is also of particular interest to health psychologists given the centrality of attitudinal variables in many core psychological models (e.g. Theory of Planned Behaviour, Health Belief Model). The capacity to graphically visualise complex patterns of relationships further offers the potential for insight into the salient psychological processes and to highlight theoretical gaps. For example, Langley, Wijn, Epskamp, and Van Bork (2015) used network analysis to examine the Health Belief Model variables in relation to girls’ intentions to obtain HPV vaccination. They reported that although some aspects of the HBM (e.g. perceived efficacy) were related to intentions, other core constructs such as cues to action were less relevant. In addition, social factors, currently not included in the HBM, were important in the network; such research can inform conceptual developments linking individual beliefs with social context to better understand healthy behaviours. Consequently, the network approach offers the potential to gain novel insights as the network structure can be analysed to reveal both core structural and relational features.

The aim of this paper is to provide an overview of networks, how they can be visualised and analysed, and to present a simple example of how to conduct network analysis on empirical data in *R* (R Core Team, 2017).

## What is a network?

At an abstract level, a network refers to various structures comprising variables, which are represented by **nodes,** and the relationships (formally called **edges**) between these nodes. For example, from the Foresight Report the variables such as stress, peer pressure, functional fitness, nutritional quality of food and drink represent nodes in the network, and the positive and negative relationships between those nodes are edges. There are some differences in nomenclature in the network literature: nodes are sometimes referred to as vertices, edges are sometimes referred to as links, and networks are also called graphs. Networks can be estimated based on cross-sectional or longitudinal time-series data; in addition, networks can be analysed at the group or individual level. Cross sectional data from a group can reveal group-level conditional independence relationships (e.g. Rhemtulla et al., 2016). Individualised networks based on times series data can provide insights into a specific individual over time (e.g. Kroeze et al., 2017). Furthermore, the networks produced by different populations can be compared. In general, network analysis represents a wide range of analytical techniques to examine different network models.

In psychological networks, nodes represent various psychological variables (e.g. attitudes, cognitions, moods, symptoms, behaviours), while edges represent unknown statistical relationships (e.g. correlations, predictive relationships) that can be estimated from the data. A node can represent a single item from a scale, a sub-scale, or a composite scale: the choice of node depends upon the type of data that provide the most appropriate and useful understanding of the questions to be addressed. Edges can represent different types of relationships, e.g. co-morbidity of psychological symptoms, correlations between attitudes.

Two types of edges can be present in a network: (1) a *directed* edge: the nodes are connected and one head of the edge has an arrowhead indicating a one-way effect, or (2) an *undirected* edge: the nodes have a connecting line indicating some mutual relationship but with no arrowheads to indicate direction of effect. Networks can be described as being directed (i.e. all edges are directed) or undirected (i.e. no edges are directed). For example, edge direction has been used in psychology networks particularly for representing cross-lagged relationships among variables (Bringmann et al., 2016). A directed network can be *cyclic* (i.e. we can follow the directed edges from a given node to end up back at that node) or *acyclic* (i.e. you cannot start at a node and end up back at that node again by following the directed edges).

Directed networks can represent causal structures (Pearl, 2000); however, such directed networks can have very strict assumptions, i.e. all the variables that have a causal effect are measured in the network, and the causal chain of cause and effect is not cyclic (i.e. a variable cannot cause itself via any path) (Epskamp, Borsboom, & Fried, 2018a). Although Directed Acyclic Graphs (DAGs) have been frequently reported in the epidemiological research literature in the past two decades (Greenland, Pearl, & Robins, 1999), the acyclic assumption may be untenable in many contexts for psychology. For example, in many psychological phenomena, reciprocal effects may exist between variables: having a positive attitude towards a behaviour results in that behaviour, which then results in a more positive attitude. In addition, directed networks suffer from the problem, similar to that arising in Structural Equation Modelling, that many equivalent models can account for the pattern of relationships found in the data (Bentler & Satorra, 2010; MacCallum, Wegener, Uchino, & Fabrigar, 1993). In their recent review of the challenges for network theory and methodology in psychopathology, Fried and Cramer (2017) note that despite the plausibility of many causal psychopathological symptom pathways in networks, there is a need to build stronger cases for the causal nature of these relationships. They highlight that many network papers have estimated undirected networks in cross-sectional data, and that even those that use directed networks based on time-series data at best show that variables measured at one moment in time can predict another variable at a different measurement time (*Granger causality*; Granger, 1969), which satisfies the requirement for putative causes preceding their effects (Epskamp et al., 2018b). Although such a temporal relationship may indicate a causal relationship, it is possible that the link may occur for other reasons (e.g. a unidimensional autocorrelated factor model would lead to every variable predicting every other variable over time; Epskamp et al., 2018b). Spirtes, Glymour, and Scheines (2000) developed the PC algorithm, which can be used to examine networks to find candidate causal structures that may have generated the observed patterns of relations present. However, such approaches have not been widely used to date in psychological networks. In general, network analysis can be considered as hypothesis-generating for putative causal structures that require empirical validation.

Edges convey information about the direction and strength of the relationship between the nodes. The edge may be ***positive*** (e.g. positive correlation/covariance between variables) or ***negative*** (e.g. negative correlation/covariance between variables); the polarity of the relationships is represented graphically using different coloured lines to represent the edges: positive relationships are typically coloured blue or green, and negative relationships are coloured red. Edges can be either ***weighted*** or ***unweighted***. A weighted edge reflects the strength of the relationship between nodes by varying the thickness and colour density of the edge connecting the nodes: thicker denser coloured lines indicate stronger relationships. Alternatively, the edge may be unweighted and simply represent the presence *vs*. absence of a relationship; in such a network, the absence of a relationship results in the nodes not having a connecting edge.

**Figure 1.**
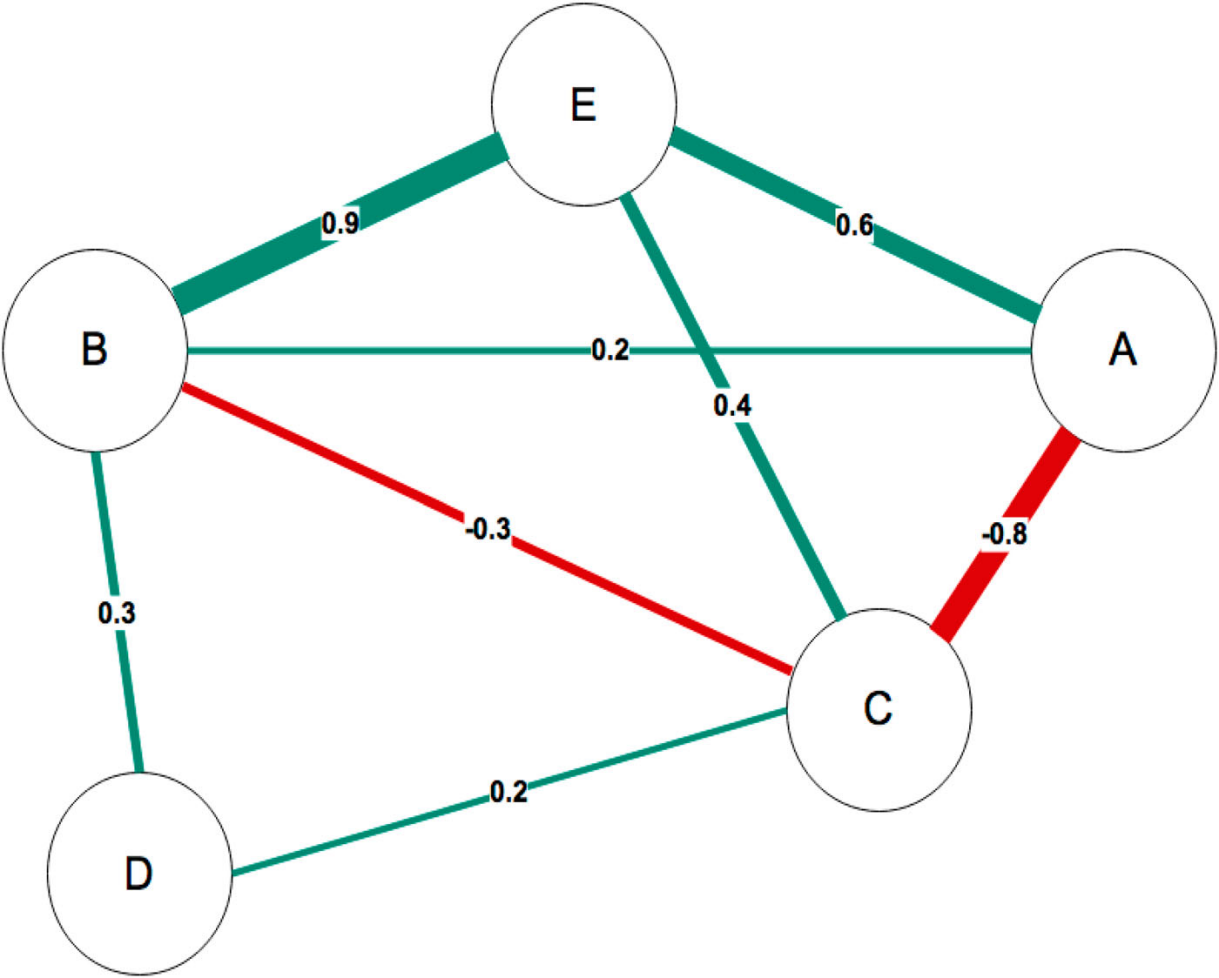
Sample network with 5 nodes and 8 edges. Postive edges are green and negative edges are red. The numbers represent the correlations between the variables.

Figure 1 presents a simple network model representing the partial correlation matrix between 5 variables (A - E) below (Table 1). The size and colour density of the lines (edges) vary to reflect the varying strength of relationship between the variables; the edges are non-directional as the data represented as bivariate partial correlations between the variables. The network comprises both positive (green lines) and negative correlations (red lines) between the variables. Some variables are more central and have more connections than others: C relates to all the variables in the network, whereas D only relates to two other variables.

**Table 1.** Partial correlation matrix between 5 variables.

Variable	*A*	*B*	*C*	*D*
*B*	.2	–	–	–
*C*	−.8	−.3	–	–
*D*	0	.3	.2	–
*E*	.6	.9	.4	0

Having briefly outlined the basic features of a network, the next sections will outline the three core analytical steps in network analysis:

1. Estimate the network structure based on a statistical model that reflects the empirical patterns of relationships between the variables
2. Analyse the network structure
3. Assess the accuracy of the network parameters and measures.

*1. Estimating the Network*

Historically, network science has developed using graphical approaches to represent relationships between nodes. For example, Leonhard Euler’s application of ‘geometry of position’, Gustav Kirchoff’s work on the algebra of graphs in relation to electrical networks, and Cayley’s contributions to molecular chemistry all utilised graphical approaches to network data (Estrada & Knight, 2015). The network visually represents the pattern of relationships between variables and a network can be estimated using common statistical parameters that quantify relationships, e.g. correlations, covariances, partial correlations, regression coefficients, odds ratios, factor loadings. However, as correlation networks can contain spurious edges, for example due to an (unmeasured) confounding variable, the most common approach in psychology uses partial correlations to create the relationships between variables. For example, if we had a network examining the relationship between risk behaviours (e.g. caffeine consumption) and health outcome (e.g. cancer), the analysis would show a relationship between the variables; however, such a relationship may simply reflect the fact that an unmeasured confound (e.g. smoking) is associated with both caffeine consumption and cancer. Partial correlations, similar to multiple regression coefficients, provide estimates of the strength of relationships between variables controlling for the effects of the other measured variables in the network model. Thus it is critically important to measure such potential confounding variables to ensure that their effects are controlled for. Two nodes are connected if there is covariance between those nodes that cannot be explained by any other variable in the network. The resulting partial correlations not only provide an estimate of the direct strength of relationships, but can also indicate mediation pathways: in Figure 1 A and D are not directly connected (i.e. no edge between them) but A influences C, which in turn influences D, thus C mediates the relationship between A and D. Partial correlation networks can provide valuable hypothesis generating structures, which may reflect potential causal effects to be further examined in terms of conditional independence (Pearl, 2000).

As noted previously, undirected network models in psychology have typically been examined, and a frequently used model in estimating such networks is the pairwise Markov Random Field (PMRF), which is a broad class of statistical models. A PMRF model is characterised by undirected edges between nodes that indicate conditional dependence relations between nodes. An absent edge means that two nodes are conditionally independent given all other nodes in the network. An edge indicates conditional dependence given all other nodes in the network. Different PMRF models can be used, depending upon the type of data (continuous, ordinal, binary, or mixtures of these data types) to be modelled. When continuous data are multivariate normally distributed, analysing the partial correlations using the *Gaussian graphical model* (GGM; Costantini et al., 2015; Lauritzen, 1996) is appropriate. If the continuous data are not normally distributed then a transformation (e.g. nonparanormal transformation, Liu, Lafferty, & Wasserman, 2009) can be applied prior to applying the GGM. The GGM can also be used for ordinal data, wherein the network is based on the polychoric correlations instead of partial correlations (Epskamp, 2018). If all the research variables are binary, the *Ising Model* can be used (van Borkulo et al., 2014). When the data comprise a mixture of categorical and continuous variables, the *Mixed Graphical Model* can be used to estimate the PMRF (Haslbeck & Waldorp, 2016). Thus, networks can be estimated from various types of data in a flexible manner.

The network complexity requires consideration. The higher the number of nodes being examined, then the higher the number of edges have to be estimated: in a network with five nodes, 10 unique edges are estimated, whereas in a network with 10 nodes, 45 edges are estimated, and in a network with 20 nodes, 190 edges are estimated. In addition, in the case of an Ising model not only are edge weights estimated but so too are thresholds: in the case of 20 nodes that would mean an additional 20 parameters to be estimated. However, as mentioned above many of these edges (e.g. correlations) may be spurious, and an increase in the number of nodes can lead to over-fitting and very unstable estimates (Babyak, 2004). Like all statistical techniques that use sample data to estimate parameters, the correlation and partial correlations values will be influenced by sample variation and therefore exact zeros will be rarely observed in the matrices. Consequently, correlation networks will nearly always be fully connected networks, possibly with small weights on many of the edges that reflect weak and potentially spurious partial correlations. Such spurious relationships will be problematic in terms of the network interpretation and will compromise the potential for network replication. In order to limit the number of such spurious relationships, a statistical regularisation technique, which takes into account the model complexity, is frequently used.

A ‘least absolute shrinkage and selection operator’ (LASSO; Friedman, Hastie, & Tibshirani, 2008) with a tuning parameter set by the researcher is applied to the estimation of the partial correlation networks. The LASSO performs well in the estimation of partial correlation networks (Fan, Feng, & Wu, 2009), and it results in some small weak edge estimates being reduced to exactly zero, resulting in a sparse network (Tibshirani, 1996). The LASSO yields a more parsimonious graph (fewer connections between nodes) that reflects only the most important empirical relationships in the data. Of note, the absence of an edge does not present evidence that the edge is in fact exactly zero (Epskamp, Kruis, Marsman, & Marinazzo, 2017). The goal of the LASSO is to exclude spurious relationship but in doing so, it may omit actual relationships. Although many variants of the LASO have been developed, the graphicalLASSO (*glasso*, Friedman et al., 2008) is recommended both in terms of ease of implementation in specific analysis programmes but also its felxibility in terms of non-continuous data (Epskamp & Fried, In Press). The edge may be absent from the network if the data are too messy and noisy to detect the true relationship, and quantifying evidence for edge weights being zero is an ongoing research issue (Wetzels & Wagenmakers, 2012). Simulation studies show that the LASSO has a low likelihood of false positives, which provides some confidence that an observed edge is indeed present in the network (Krämer, Schäfer, & Boulesteix, 2009). However, the specific nature of the relationship reflected in the edge is still uncertain, e.g. the edge could represent a direct causal pathway between nodes, or it could reflect the common effect of a (latent) variable not included in the network model.

As mentioned previously, the use of the LASSO requires setting a tuning parameter. The sparseness of the network produced using the LASSO depends upon the value the researcher sets tuning parameter (λ): the higher the λ value selected the more edges are removed from the network and its value directly influences the structure of the resulting network. The tuning parameter λ therefore needs to be carefully selected to create a network structure that minimises the number of spurious edges while maximising the number of true edges (Foygel & Drton, 2010). In order to ensure that the optimal tuning parameter is selected, a common method involves estimating a number of networks under different λ values. These different networks range from a completely full network where every node is connected to each other to an empty network where no nodes are connected. The LASSO estimates produce a collection of networks rather than a single network; the researcher needs to select the optimal network model and typically this is achieved by minimising the Extended Bayesian Information Criterion (EBIC; Chen & Chen, 2008), which has been shown to work particularly well in identifying the true network structure (Foygel & Drton, 2010; van Borkulo et al., 2014), especially when the true network is sparse. Model selection using the EBIC works well for both the Ising model (Foygel Barber & Drton, 2015) and the GGM (Foygel & Drton, 2010). The EBIC has been widely used in psychology networks (e.g. Beard et al., 2016; Isvoranu et al., 2017) and it enhances both the accuracy and interpretability of networks produced (Tibshirani, 1996).

The EBIC uses a hyperparameter (*γ*) that dictates how much the EBIC will prefer sparser models (Chen & Chen, 2008; Foygel & Drton, 2010). The *γ* value is determined by the researcher and is typically set between 0 and 0.5 (Foygel & Drton, 2010), with higher values indicating that simpler models (more parsimonious models with fewer edges) are preferred. In many ways the choice of *γ* depends upon the extent to which the researcher is taking a liberal or conservative approach to the network model. A value of 0 results in more edges being estimated, including possible spurious ones, but which can be useful in early exploratory and hypotheses generating research. Of note, a *γ* setting of zero will still produce a network that is sparser compared to a partial correlation network that has not be regularised using a LASSO. Although *γ* can be set at 1, the default in many situations is 0.5. Foygel and Drton (2010) suggest that setting the *γ* value 0.5 will result in fewer edges being retained, which will remove the spurious edges but it may also remove some other edges too. A compromise value *γ* of 0.25 is potentially a useful value to also use to see the impact on the network model produced.

**Figure 2.**
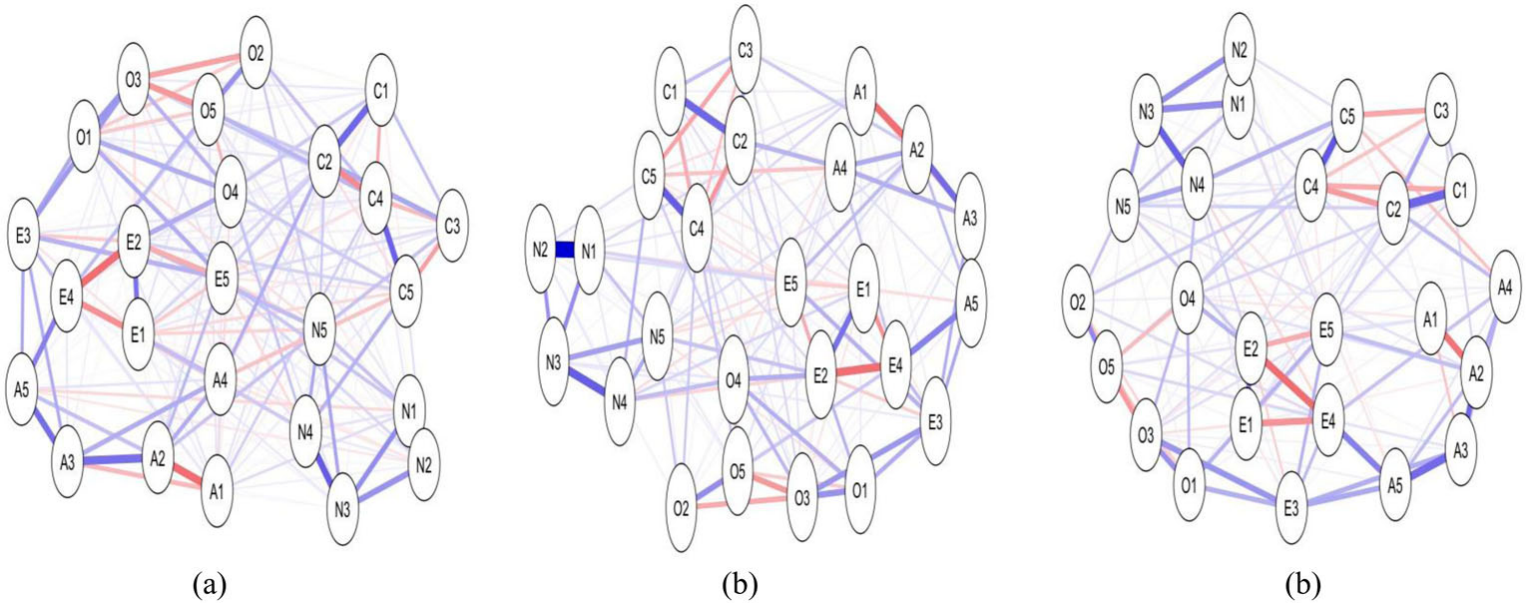
Partial correlation networks estimated on same dataset, with increasing levels of the LASSO hyperparameter *γ* (from left to right: Panel (a) *γ* = 0, Panel (b) *γ* = 0.5, Panel (c) = 0.99).

Figure 2 presents the same data (questionnaire items on the big 5 model of personality, with 5 items for each dimension: Openness, Conscientiousness, Agreeableness, Extraversion, and Neuroticism) analysed using *γ* of 0, 0.5, and 0.99. With the tuning parameter set to 0, the network contains a dense array of connections as more edges are estimated; as the tuning parameter increases, the number of edges estimated decreases as the model become more sparse. This illustrates that the choices made by the researchers in setting the *γ* level will impact on the nature of the network produced. Of note, Epskamp and Fried (In Press) report that comparison of networks based on simulated data using *γ* of 0.00, 0.25 and 0.50 revealed the higher values of *γ* were able to reveal the true network structure but that the value of 0 included a number of spurious relationships. They caution that *γ* of .5 may still be conservative and not reflect the true model, and they note that the choice of *γ* is somewhat arbitrary and up to the researcher. Epskamp (2018) reported recently that increasing the *γ* to 0.75 or 1.00 did not outperform a *γ* of 0.5 in a well-established personality dataset.

In order to plot the network, the nodes and edges need to be positioned in manner that reflects the patterns of relationships present in the data. The most frequently used approach in psychological networks is the Fruchterman-Reingold algorithm (Fruchterman & Reingold, 1991), which calculates the optimal layout so that nodes with less strength and less connections are placed further apart, and those with more and/or stronger connections are placed closer to each other. The development of qgraph as a package to visualise patterns of relationships between nodes in networks was an invaluable contribution to advancing network analysis (Epskamp, Cramer, Waldorp, Schmittmann, & Borsboom, 2012).

*2. Network Properties*

After a network structure is estimated, the graphical representation of the network reveals the structural relationships between the nodes, and we can then further analyse the network structure in terms of its properties. This analysis provides insight into critically important features of the network. For example, are certain nodes more important (central) than others in the network? Is the global structure dense or sparse? Does it contain strong clusters of nodes (communities) or are the nodes isolated?

## Centrality

Not all nodes in a network are equally important in determining the network’s structure: centrality indices provide insight into the relative importance of a node in the context of the other nodes in the network (Borgatti, 2005; Freeman, 1978). For example, a central symptom is one that has a large number of connections in a network and its activity can spread activation throughout a symptoms network; in contrast, a peripheral symptom is on the outskirts of a network and has few connections and consequently less impact on the network. Different centrality indices provide insights into different dimensions of centrality. The indices can be presented as standardised z score indices to provide information on the *relative* importance of the nodes, and judging centrality requires careful consideration of the different dimensions in combination. These indices are based on the pattern of the connections in which the node of interest plays a role and can be used to model or predict several network processes, such as the amount of flow that traverses a node or the tolerance of the network to the removal of selected nodes (Borgatti, 2005). The most common aspects of centrality typically examined are as follows.

*Degree**:*** degree centrality is defined as the number of connections incident to the node of interest (Freeman, 1978).

*Node strength*: how strongly a node is directly connected to other nodes is based on the sum of the weighted number and strength of all connections of a specific node relative to all other nodes. Whilst degree provides information on the number of connections, strength can provide additional information on the importance of that node, for example a node with many weak connections (high degree) might not be as central to the network as one that has fewer but stronger connections. However, as noted by Opsahl, Agneessens, and Skvoretz (2010) merely focusing on node strength alone as an index of importance is potentially misleading as it does not take account of the number of other nodes to which it connected. Consequently, it is important to incorporate both degree and strength as indicators of the level of involvement of a node in the surrounding network when examining the centrality of a node. Opsahl et al. (2010) proposed the use of a degree centrality measure, which is the product of the number of nodes that a specific node is connected to, and the average weight of the edges to these nodes adjusted by an alpha (*α*) parameter, which determines the relative importance of the number of edges compared to edge weights. In combining both degree and strength, the tuning *α* parameter is set by the researcher: if this parameter is between 0 and 1, then having a high degree is regarded as favourable, whereas if it is set above 1, then a low degree is favourable.

*Closeness*: the closeness index quantifies the node’s relationship to all other nodes in the network by taking into account the indirect connections from that node. A high closeness index indicates a short average distance of a specific node to all other nodes; a central node with high closeness will be affected quickly by changes in any part of the network and can affect changes in other parts of the network quickly (Borgatti, 2005).

*Betweenness*: the betweenness index provides information on how important a node is in the average pathway between other pairs of nodes. A node can play a key role in the network if it frequently lies on the shortest path between two other nodes, and it is important in the connection that the other nodes have between them (Saramäki, Kivelä, Onnela, Kaski, & Kertész, 2007; Watts & Strogatz, 1998).

*Clustering**:*** the extent to which a node is part of a cluster of nodes can be estimated (Saramäki et al., 2007). The local clustering coefficient *C* is the proportion of edges that exist between the neighbours of a particular node relative to the total number of possible edges between neighbours (Bullmore & Sporns, 2009). It provides insight into the local redundancy of a node: does removing the node have an impact on the capacity of the neighbouring nodes to still influence each other? An overall global clustering coefficient (also referred to as transitivity) for the entire network can be estimated in both undirected and directed networks. Furthermore, the overall network may comprise *communities*, i.e. a clustering of nodes that are highly interconnected among themselves and poorly connected with nodes outside that cluster.

Detecting communities requires researchers to not simply interpret the placement of nodes in the visual representation of the data but to examine the patterns present using a formal statistical approach. Fried (2016) highlights a number of approaches to help identify communities. As latent variable models and network models are mathematically equivalent, examining the eigenvalues of components present in data using exploratory factor analysis is one way to identify how many communities might be present and the factor loadings indicate which nodes belong to which community. More sophisticated approaches include the *spinglass algorithim* (although this is limited by the fact that it often produces different results every time you run it, and it only allows nodes to be part of one community, whereas nodes may be better described as belonging to several communities at the same time), the *walktrap algorithim* (which provides more consistent results if you repeat it, but which also only allows nodes to be part of one community), and the Clique Percolation Method (CPM), which allows nodes to belong to more than one community (see Blanken et al., 2018).

## Overall network topology

Networks can take on many different shapes; however, some common network shapes have been described in detail in the literature. Random networks comprise nodes with random connections, with each node have approximately the same number of connections to others. The distribution of the nodes’ connections follows a bell-curve. ‘Small world’ networks are characterised by relatively high levels of transitivity and nodes being connected to each other through small average path lengths (Watts & Strogatz, 1998). A classic example of the ‘small-world effect’ is the so-called ‘six degrees of separation’ principle, suggested by Milgram (1967). Letters passed from person to person reached a designated target individual in only a small (approximately 6) number of steps; the nodes (individuals) were connected by a short path through the network.

‘Scale free’ networks are characterised by a relatively small number of nodes that are connected to many other nodes (Barabási, 2012). These ‘hub’ nodes have an exceptionally high number of connections to other nodes, whereas the majority of non-hub nodes have very few connections. The distribution of the nodes’ connections follows a power law. Research has found that HIV transmission among men who have sex with men can be modelled as a scale free model (Leigh Brown et al., 2011); identifying individuals who are have very high levels of connections and represent ‘*superspreaders*’ of infections provides an efficient means for targeted vaccinations (Pastor-Satorras & Vespignani, 2001). Within scale free networks, nodes with high centrality measures and extremely higher centrality than other nodes may be ‘hubs’. However, it is critically important to check the pattern of directed relationships between the node and its neighbours, e.g. in a directed network a node could have a high centrality because it has many directed edges to other nodes (high OutDegree centrality) whilst having no edges from those nodes pointing at it (zero InDegree centrality); in this case the node would not be a hub.^1^

In addition to group-level analysis, networks can be developed at a person-specific level: a time-series network of an individual may be useful for understanding the relationship between nodes (e.g. symptoms) at an individualised level, and could be used for personalised treatment planning (David, Marshall, Evanovich, & Mumma, 2018). If network structures are replicated and nodes emerge as hubs, then changing these hub nodes might have downstream effects on other nodes, which might result in an efficient means to change outcomes (Isvoranu et al., 2017). For example, network analysis may reveal that a certain belief is a hub and therefore critical in terms of impact on behaviour change: therefore we could focus our efforts on changing that belief rather than attempting to change multiple beliefs. Developing a better understanding of the structural relationships between the nodes in the network can provide important theoretical and practical insights for health psychology.

*3. Network accuracy*

As the network is based on sample data, the accuracy of the sample-based estimates of the population parameters reflecting the direction, strength and patterns of relationships between nodes should be considered. To-date much of the research on networks has used edge strength and node centrality to make inferences about the phenomenon being modelled. However, as Epskamp et al. (2018a) note, relatively little attention has been paid towards examining the accuracy of the edge and centrality estimates. Given the relatively small sample sizes that typically characterises psychological research, edge strengths and node centrality may not be estimated accurately. Therefore, it is recommended that researchers determine the accuracy of both. The accuracy of edge weights is estimated by calculating confidence intervals (e.g. 95% CI) for their estimates. As a CI requires knowledge of the sampling distribution of the estimate, which may be difficult to obtain for the edge weight estimate, Epskamp et al. (2018a) developed a method that uses bootstrapping (Efron, 1979) to repeatedly estimate a model under either sampled or simulated data, and then estimates the required statistic. The more bootstrap samples that are run, the more consistent the results. Either a parametric bootstrap or non-parametric bootstrap can be applied for edge-weights (Bollen & Stine, 1992). For non-parametric bootstrapping, observations in the data are resampled with replacement to create new plausible datasets. Parametric bootstrapping samples new observations from the parametric model that has been estimated from the original data; this creates a series of values that can be used to estimate the sampling distribution. Consequently, the parametric bootstrap requires a parametric model of the data whereas the non-parametric bootstrap can be applied to continuous, categorical and ordinal data. As the non-parametric bootstrap is data-driven and less likely to produce biased estimates with LASSO regularised edges (which tend to dominate in the literature), Epskamp et al. (2018a) emphasise the usefulness and general applicability of the non-parametric bootstrap. If the bootstrapped CIs are wide, it becomes hard to interpret the strength of an edge.

The accuracy of the centrality indices can be examined by using a different type of bootstrapping: subsets of the data are used to investigate the stability of the order of centrality indices based on the varying sub-samples (*m out of n* bootstrap; Chernick, 2011). The focus is on whether the order of centrality indices remains the same after re-estimating the network with less cases or nodes. A case-dropping subset bootstrap can applied and the correlation stability (CS) coefficient can quantify the stability of centrality indices using subset bootstraps. The correlation between the original centrality indices (based on the full data) is compared to the correlation obtained from the subset of data representing different percentages of the overall sample. For example, what is the correlation between the estimates from the entire data with the estimates based on a subset of 70% of the original sample? A series of such correlations can be presented to illustrate how the correlations change as the subset sample gets smaller (95% of the sample, 80%, 70%, … .25%). If the correlation changes considerably, then the centrality estimate may be problematic. A correlation stability coefficient of .7 or higher between the original full sample estimate and the subset estimates has been suggested as being a useful threshold to examine (Epskamp et al., 2018a). A *CS*-coefficient (correlation = .7) represents the maximum proportion of cases that can be dropped, such that with 95*%* probability the correlation between original centrality indices and centrality of networks based on subsets is 0.7 or higher (Epskamp et al., 2018a). It is suggested that the *CS*-coefficient should not be below 0.25, and preferably it should be above 0.5.

### Other applications of network analysis

The majority of research has examined networks based on cross-sectional data from a single group of participants. However, networks can also be determined for individuals over time as well as for comparing different groups. A network can be created for an individual based on time-series data to provide insights into that specific individual. Nodes that are identified as hubs in such networks could be important targets for interventions (Valente, 2012). Networks can be developed that model temporal effects between consecutive data measurements. The graphical VAR model (Wild et al., 2010) uses LASSO regularisation based on BIC to select the optimal tuning parameter (Abegaz & Wit, 2013). When multiple individuals are measured over time, multi-level VAR can be used and it estimates variation due to both time and to individual differences (Bringmann et al., 2013).

Networks can be estimated for different groups. Although the lack of methods comparing networks from different groups has been noted (Fried & Cramer, 2017), joint estimation of different graphical models (Danaher, Wang, & Witten, 2014; Guo, Levina, Michailidis, & Zhu, 2011) may prove useful in this context. For example the Fused Graphical Lasso (FGL) was recently used to compare the networks of borderline personality disorder patients with those from a community sample (Richetin, Preti, Costantini, De Panfilis, & Mazza, 2017). In addition, van Borkulo and colleagues have developed the Network Comparison Test (NCT) to allow researchers to conduct direct comparisons of two networks as estimated in different subpopulations (Van Borkulo, 2018). The test uses permutation testing in order to compare network structures that involve relationships between variables that are estimated from the data. The test focuses on the extent to which groups may differ in relation to (1) the structure of the network as a whole, (2) a given edge strength, (3) and the overall level of connectivity in the network. For example, research has reported that the network of MDD symptoms for those with persistent depression was more strongly connected than the network of those with remitting depression (van Borkulo et al., 2015).

## Network analysis issues

Like all statistical models, the network model represents an idealised version of a real-world phenomenon that we wish to understand. In selecting the variables to be modelled we must decide which variables to include and how they are to be measured: each of these processes introduces error into the modelling process. A general concern for networks concerns their replicability (e.g. see Forbes, Wright, Markon, & Krueger, 2017; and responses by Borsboom et al., 2017; Steinley, Hoffman, Brusco, & Sher, 2017) and research needs to address this issue by estimating the stability of the networks and examining generalizability of the network model. As noted by Fried and Cramer (2017) the literature in general requires more conceptual and methodological developments for estimating both the accuracy and stability of networks. The identification of useful thresholds for these parameters will also prove critical in the interpretation of the network models. Similar to other methods of analysis (e.g. regression, SEM), network analysis is sensitive to the variables in the model and to the specific estimation methods used. Hence, the challenges regarding replication and generalizability are not unique to network modelling.

The larger the sample size, the more stable and accurately networks are estimated. Given the recent growth in use network analytic approaches in psychology it is not easy to hypothesise expected network structure and edge weights, which means there is little evidence to guide *a priori* power analyses. Epskamp et al. (2018a) note that as more network research is conducted in psychology, more knowledge will accumulate regarding the nature of network structure and edge-weights that can be expected.

The dominant methods to date used to discover network structures in psychology are based on correlations, partial correlations, and patterns of conditional independencies. Further developments and application of causal model techniques will advance understanding of the relationships present in networks (Borsboom & Cramer, 2013). As noted previously, much of the research in psychological networks has been based on exploratory data analyses to generate networks; there is a need to progress towards confirmatory network modelling wherein hypotheses about network structure are formally tested.

## How to run network analysis: an example using *R*

Many network structure analysis methods can be implemented in the generic software MATLAB and Stata, or specialised network software packages including UCINET (Borgatti, Everett, & Freeman, 2002) or Gephi (https://gephi.org). The Stanford Network Analysis Platform (SNAP) provides a network analysis library. *R* is an open-source statistical programming language that facilitates statistical analysis and data visualisation (R Core Team, 2017); to date much of the research on psychological networks has used *R*-packages igraph (Csárdi & Nepusz, 2006) or qgraph (Epskamp et al., 2012). Of note, the psychosystems research group has created specific *R* packages that make network analysis easier to implement (see *psychosystems.org)*. As mentioned at the start of this paper, their website is an essential resource for conducting network analysis in psychology. In this example, we will use the *bootnet* package as it provides a comprehensive suite of analytical options for network analysis. Data can inputted straight into *R* or can be imported in various common formats (e.g. csv. or txt. file) or from other data analysis programmes, e.g. Excel, SPSS, SAS and Stata.

*R* can be obtained via the https://www.r-project.org/ webpage. To download *R*, you need to select your preferred CRAN (Comprehensive *R* Archive Network) mirror (https://cran.r-project.org/mirrors.html). On the Mirrors webpage, you will find listings of countries that have identical versions of *R* and should select a location geographically close to your computer’s location. *R* can be downloaded for Linux, Windows, and Mac OS. The pages are regularly updated and you need to check with releases are supported for your platform. *R* as a base package can perform many statistical analyses but most importantly, *R*’s functionality can be expanded by downloading specific packages.

After installing *R* (https://www.r-project.org/), it is quite useful to also install *R* Studio (https://www.rstudio.com/), which provides a convenient interface to *R*. Once both are installed, opening up *R* Studio will give a window that is split into 4 panes:

*Console/Terminal*: this pane is the main graphical interface for the user and this is where the commands are typed in.

*Editor*: this pane shows the active datasets that you are working on.

*Environment/History/Connections*: this pane shows the *R* datasets and allows you to import data from text (e.g. csv. file), Excel, SPSS, SAS and Stata. The History tab allows you see the list of your previous commands.

*Files/plots/packages/help:* this pane and its tabs can open files, view the most current plot (also previous plots), install and load packages, or use the general *R* help function.

Under the Tools drop down tap at the top of the *R* Studio screen, you can select which packages to install for the analyses required. Alternatively the packages can be installed using the Packages tab or they can be directly installed using a typed command. *R* is a command line driven programme and you can enter commands at the prompt (> by default) and each command is executed one at a time. For the current example, you will need to install 2 packages (‘ggplot2’ and ‘bootnet’) and the relevant command lines are:

>Install.packages("ggplot2")

>Install.packages("bootnet")

Once installed, the packages need to be loaded into *R* using the library("name of package") command.

>library("ggplot2")

>library("bootnet")

Next we need to tell *R* to import the data, in this case a csv. file called TPB2018.

**Figure 3.**
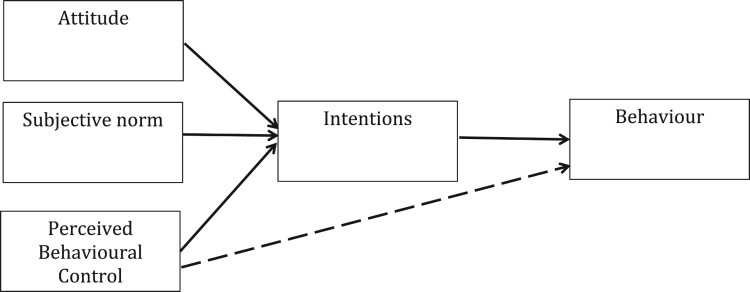
Theory of planned behaviour.

The data are taken from a study conducted using the Theory of Planned Behaviour (TPB; Ajzen, 1985, 2011). The TPB assumes that volitional human behaviour is a function of (1) one’s intention to perform a given behaviour and (2) one’s perception of behavioural control (PBC) regarding that behaviour (Figure 3). Furhermore, intentions are influened by one’s attitudes towards the behaviour (e.g. *cognitive attitudes*: is the behaviour good or bad?; *affective attitudes*: is the behaviour pleasant or unpleasant?), one’s subjective norm beliefs (e.g. *descriptive norms*: do others perform the behaviour?; *injunctive norms*: do others who are important to me want me to perform the behaviour?), and one’s perceptions of control regarding the behaviour (e.g. *self efficacy*: level of confidence to perform the behaviour; *perceived control*: barriers to stop the behavoiur being performed). The extent to which PBC influences behaviour directly, rather than indirectly through intention, depends on the degree of actual control over performing the behaviour (Sniehotta, Presseau, & Araújo-Soares, 2014). The TPB has been a dominant theoretical approach in health behaviour research for a number of decades and has been examined extensively. The vast majority of studies have used correlational designs to investigate cross-sectional and prospective associations between TPB variables and behaviour (Noar & Zimmerman, 2005); systematic reviews indicate that the TPB accounts for approximately 20% of variannce in health behaviour, and that intention is the strongest predictor of behaviour (McEachan, Conner, Taylor, & Lawton, 2011).

Following receipt of ethical approval from the local university REC (2014/6/15), students completed a questionnaire regarding regular exercise (Datafile in supplementary material). This cross-sectional dataset is used here to illustrate how to conduct a network analysis and comprises the responses of 200 students to a TPB questionnaire, which included the following items relating to regular exercise (i.e. exercising for at least 20 min, three times per week) for the next two months:

*Att1*: belief that engaging in regular exercise is healthy

*Att2:* belief that engaging in regular exercise is useful

*Att3*: belief that engaging in regular exercise is enjoyable

*Dnorm1*: descriptive norms for friends regarding engaging in regular exercise

*Dnorm2*: descriptive norms for other students regarding engaging in regular exercise

*Injnorm1*: injunctive norms for friends regarding engaging in regular exercise

*Injnorm2*: injunctive norms for students regarding engaging in regular exercise

*Pbc1*: perceived control regarding engaging in regular exercise

*Pbc2*: self-efficacy towards engaging in regular exercise

*Intention*: intention to engage in regular exercise

In the Environment/History/Connection pane, we can select Import Dataset to import the datafile. Alternatively you can use the command code:

TPB2018 = read.csv("filename.extension", header = TRUE).

The filename extension is simply the location of the relevant csv. file on your computer.

Once it is imported, the data will appear in the Editor pane and the console window will have a line of code indicating that data is active

>View(TPB2018)

The next step is to tell *R* to estimate the network model using the EBICglasso to produce an interpretable network. The command line below tells *R* to label the results as ‘Network.’

Network <- estimateNetwork(TPB2018, default = "EBICglasso")

Once we have estimated the network, we can ask *R* to plot it.

>plot(Network, layout = "spring", labels = colnames(TPB2018))

**Figure 4.**
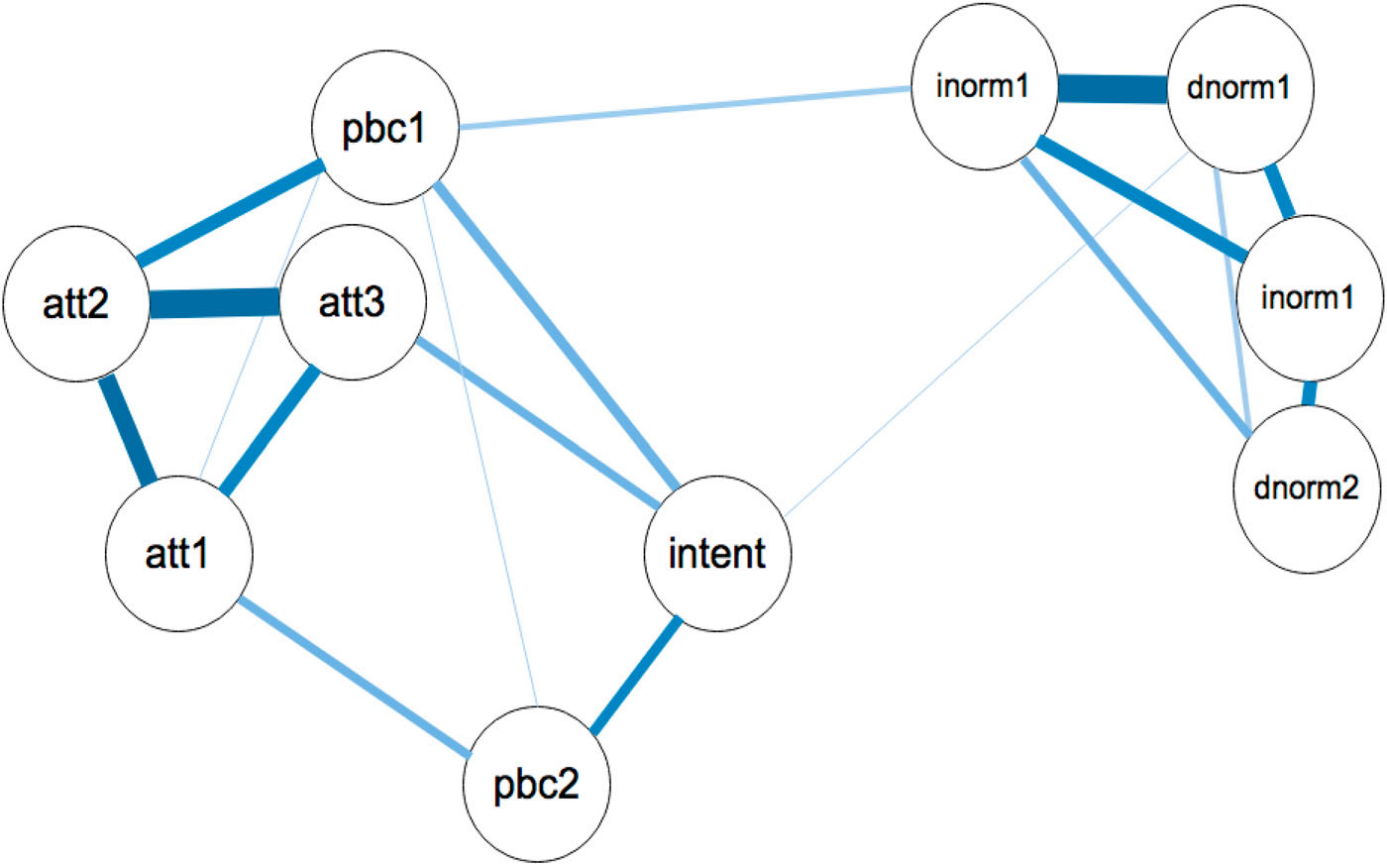
Network analysis of TPB items. The size and density of the edges between the nodes respresent the strength of connectedness.

These commands will produce the network plot with the variable names in the plot (Figure 4).

The network shows the strength of relationships between the TPB variables. Some variables have quite strong connections (e.g. *att2* and *att3*; *injnorm1* and *dnorm1*), whereas others have weak relationship (e.g. *att1* and *pbc1*). Visual inspection of the network reveals that the network seems to split into three different communities: (1) the normative beliefs cluster together; (2) the three attitudinal variables and the *pbc1* item seem to cluster, and (3) the *pbc2* and *intention* item cluster together. However, visual inspection of the graphical display of complex relationships requires careful interpretation, especially if there are a large number of nodes in the network. In order to check the presence of the potential 3 communities, a spinglass algorithm was applied to the network using the *igraph R*-package. Of note, this analysis supported the 3 community interpretation (Interested readers are referred to Eiko Fried’s tutorial on this topic: http://psych-networks.com/r-tutorial-identify-communities-items-networks/).

## Centrality

**Figure 5.**
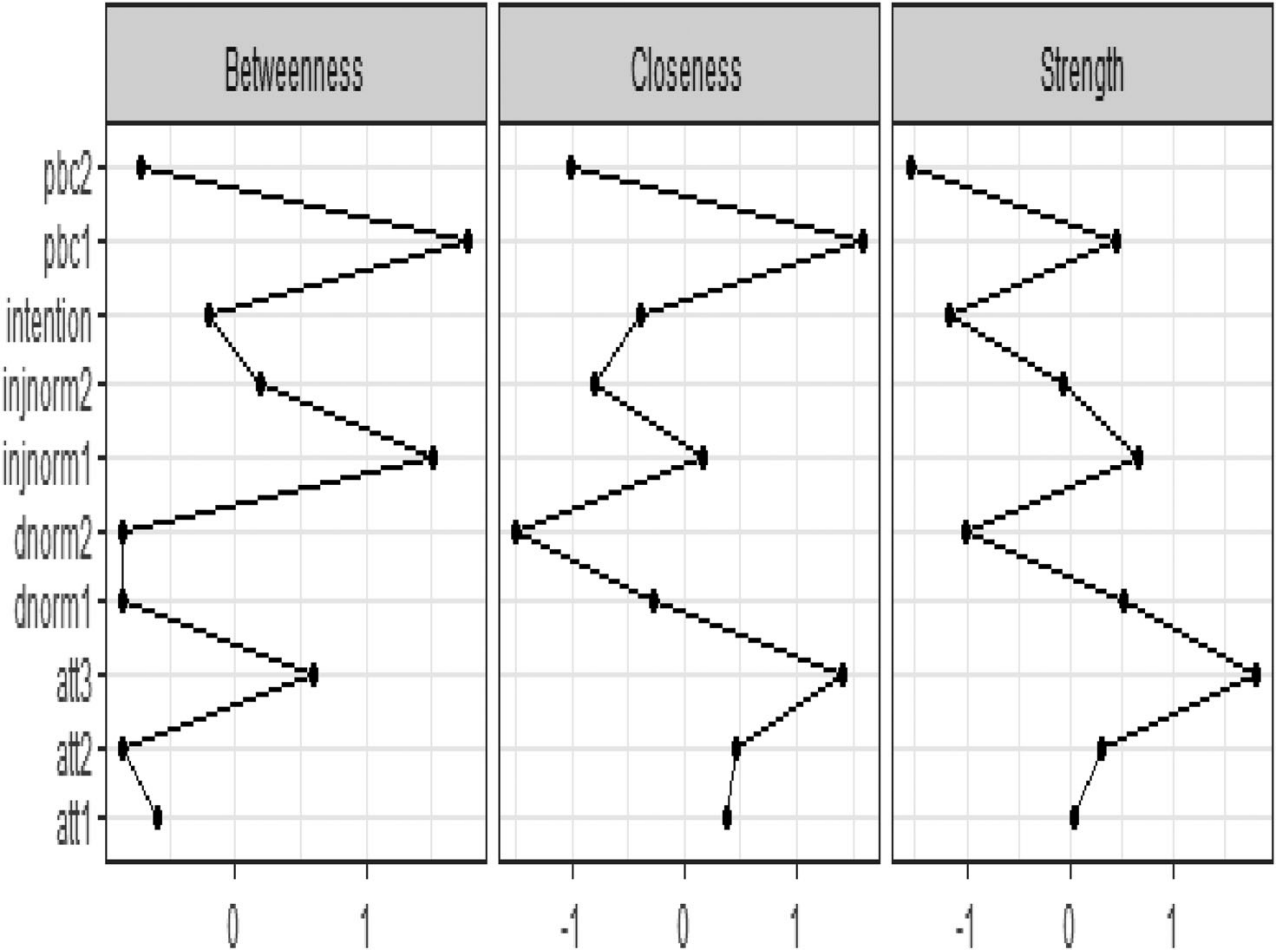
Centrality indices.

Next we can examine the centrality indices in terms of Betweenness, Closeness and Strength (Figure 5).

>centralityPlot(Network)

*Att 3* had the highest strength value and a high closeness value: it has strong connections to the nodes nearby. It plays an important role in the network and its activation has the strongest influence the other nodes in the network. However, *pbc1* and *injnorm1* had the highest betweenness values: they act as the bridge connecting the communities of nodes.

## Stability of the centrality indices

As noted previously, the stability of centrality indices can be examined by estimating network models based on subsets of the data. The case-dropping bootstrap (type = "case") is used; in this case 1000 bootstrapped samples were estimated.

>CentralStability <- bootnet(Network, nBoots = 1000, type = "case")

The CS coefficients for each index can be produced:

>corStability(CentralStability)

A table presenting summary data (e.g. *M*, *SD, CI*s) on the bootstrapped indices can be created.

>summary(CentralStability)

However, it may be more useful to plot the stability of centrality indices:

>Plot(CentralStability)

**Figure 6.**
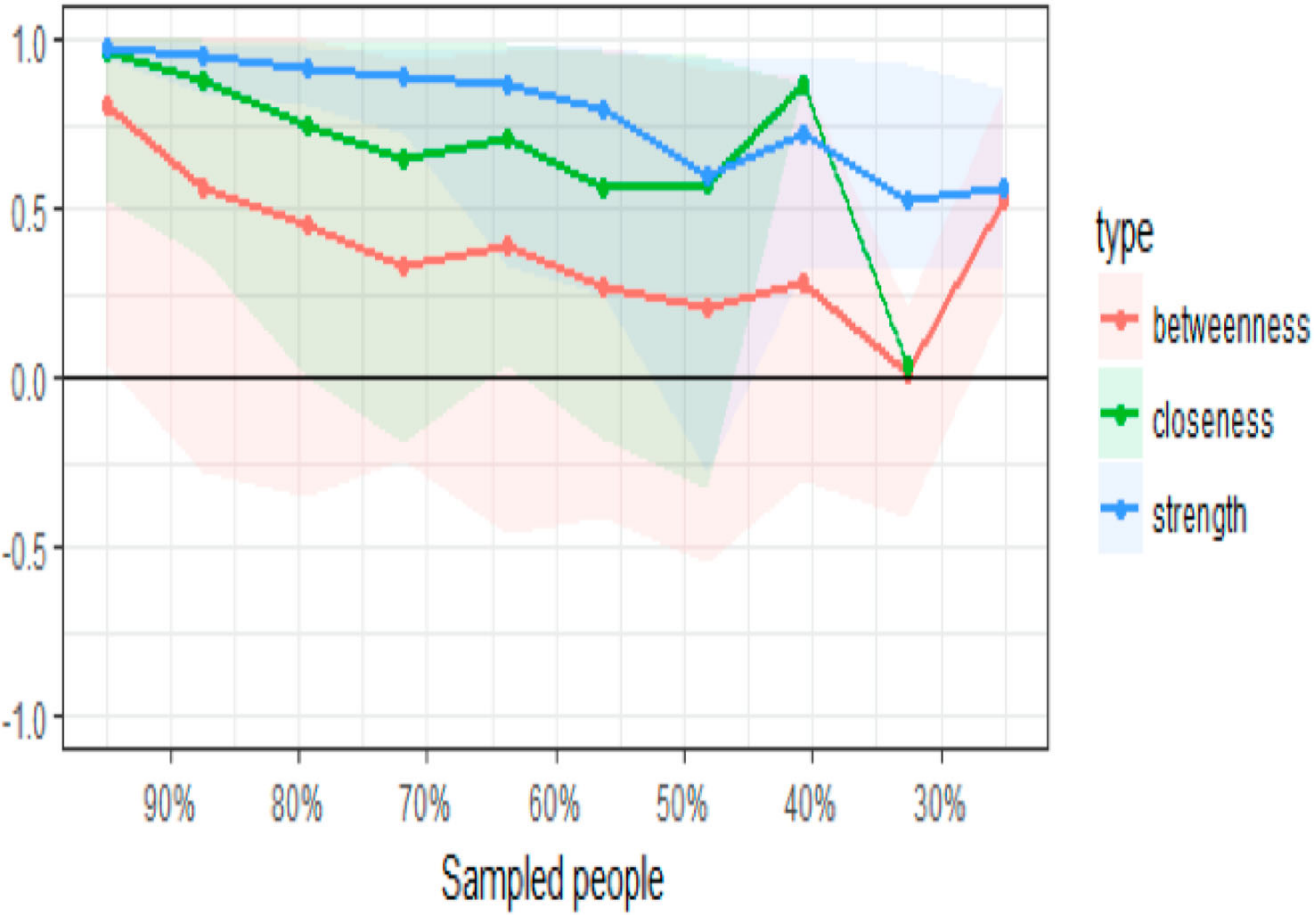
Stability of central indices.

Figure 6 shows the resulting plot of the centrality indices. As the percentage of the sample included in the estimates decreases (as illustrated on the X-axis, the subset samples decrease from 95% of the original sample to 25% of the sample), there is a drop in the correlation between the subsample estimate and the estimate from the original entire sample. Once the correlation goes below .7, then the estimates become unstable. For example, using 90% of the original sample, there is steep decrease in accuracy of the betweenness estimate, whilst the stability of the strength and closeness estimates declines at a slower rate. However, with a subset sample of 70% of the original participants, the closeness estimate is now correlating less than .7 with the full sample estimate. When the subset sample comprises 50% of the original sample, the strength estimate falls below .7. Overall, the pattern suggests the stability of the centrality indices for closeness and betweenness are not that reliable: of note, strength tends to be the most precisely estimated centrality index in psychology networks, and betweenness and closeness only reach the threshold for reliable estimation in large samples (Santos, Kossakowski, Schwartz, Beeber, & Fried, 2018).

## Edge weight accuracy

The robustness of the edge weights can be examined using bootstrapped confidence intervals.

> EdgeWgt<- bootnet(Network, nBoots = 2500)

Similar to the centrality indices, a summary table of the results of edge accuracy analysis can be produced (e.g. *M*, *SD, CI*s for estimates):

summary(EdgeWgt)

The plot of the bootstrapped CIs for estimated edge parameters provides a visually informative representation of the estimates.

> plot(EdgeWgt, labels = TRUE, order = "sample")

**Figure 7.**
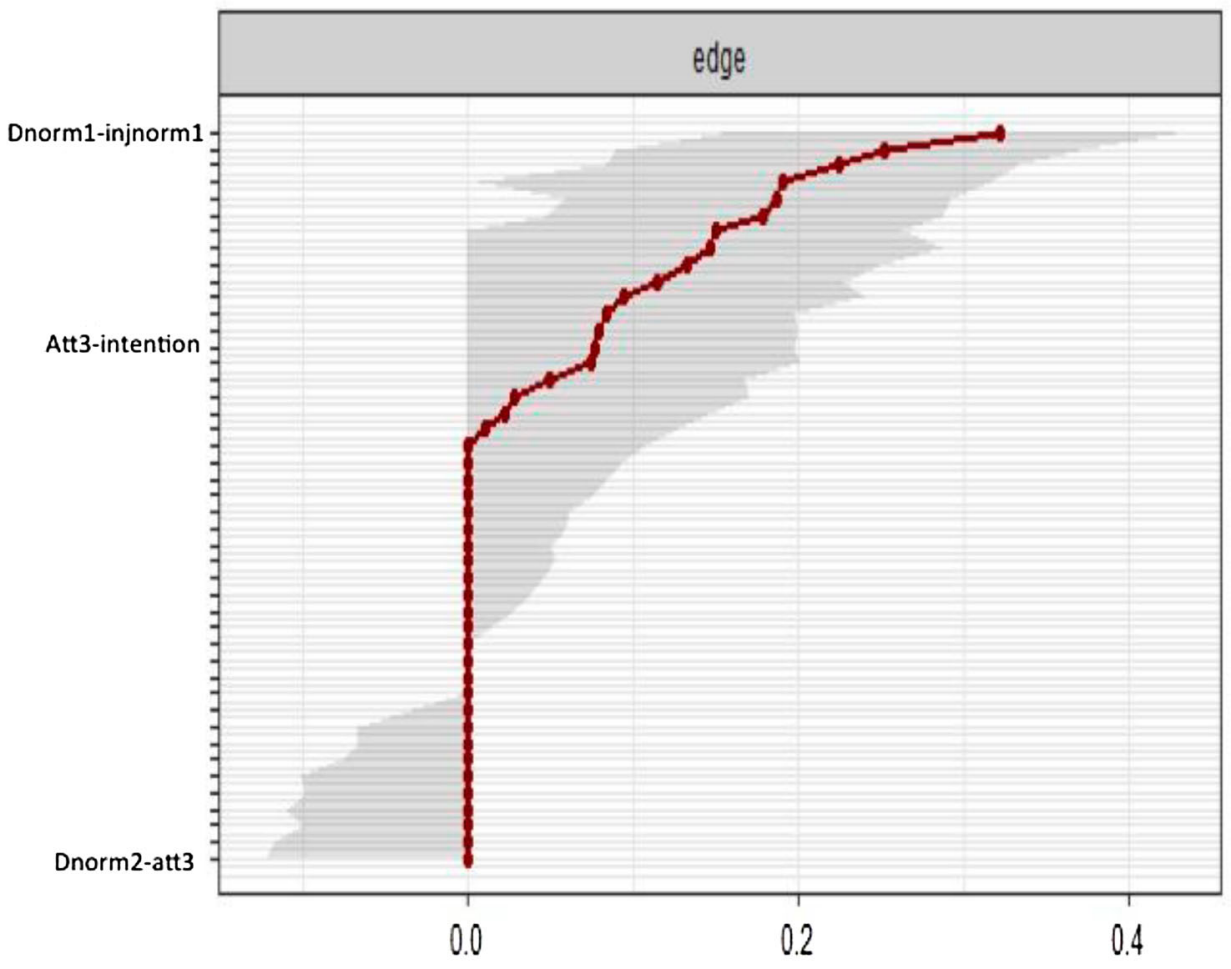
Accuracy of the edge-weight estimates (red line) and the 95% confidence intervals (grey bars) for the estimates.

Figure 7 has been modified to remove most of the names of the edges being represented on the Y axis to de-clutter the figure to enhance readability. The red line in Figure 6 shows the edge value estimated in the sample, and the grey bars surrounding the red line indicate the width of the bootstrapped CIs. Of note, many of edges are estimated as zero (e.g. *dnorm2*-*att3*). Some edges are larger then zero, but the bootstrapped CIs contain zero (e.g. *att3*-*intention*), and for a smaller number of edges, the estimates are larger than 0 and the CIs do not including zero (e.g. *dnorm1* - *injnorm1*). Given the above pattern of CIs for the edge weights, the network should be interpreted with caution.

The data were used to illustrate how to run network analysis. Typically such data are analysed by combing the items into their higher order construct (e.g. Attitudes, Norms, PBC, and Intentions) and then multiple regression examines the extent to which variation in Attitudes, Norms and PBC accounts for variation in Intentions, and which variables have significant relationships with intentions (Noar & Zimmerman, 2005). Network analysis allows us to examine how the items relate to each other and can reveal important structural relationships that regression cannot reveal. If the present network was replicated and using larger samples, then we could interpret the network in terms of its structural implications for the TPB.

Contrary to the theory, not all variables were directly related to intentions; for example att2’s (belief that exercise is useful) relationship to intention was mediated by its relationship to att1, att3 and pbc1. Indeed, all of the subjective norm items were related to intentions through a mediated pathway with pbc1. Although in line with the TPB, the normative beliefs are related to each other and form a community (i.e. the normative variables correlate with each other), in the current network, contrary to the theory, these normative beliefs have no direct relationship with intentions and only a weak relationship to PBC. This finding would indicate that your intentions to exercise are not that influenced by either the exercise behaviours of others or what you believe others would like you do in terms of regular exercise. Rather, the network suggests that your beliefs about other’s exercise only influences your perceptions of control over exercise, e.g. if others are exercising and want you to exercise, you may feel that you have more control over whether you exercise (‘if others can do it, then so can I’), and by feeling in control, you may have higher intentions to then exercise. A previous meta-analysis similarly reported lower correlations between subjective norms and intentions for physical activity behaviour compared to the strength of relationships between attitudes and intentions, and between PBC and intention (Hagger, Chatzisarantis, & Biddle, 2002).

Among the attitudinal variables, the affective attitude is the central node as it connects not only to all the other attitude variables but also to both PBC items (in line with theory) and the Intention item. Research has highlighted the role of affective attitudes on behaviour (e.g. Lawton, Conner, & McEachan, 2009) and the present data highlight the value in conceptualising normative beliefs as comprising affective/experiential and cognitive/instrumental components (Conner, 2015).

The model also found that the self-efficacy variable (pbc1) of PBC had the highest closeness to intentions; the strong relationship between self-efficacy and activity intentions is consistent with previous meta-analyses (Hagger et al., 2002). The fact that the two PBC items had differing patterns of relationships with the other TPB variables further supports the proposed distinction between the self-efficacy and perceived control components of PBC (Conner, 2015). If replicated using within person networks, the findings may suggest that changes self efficacy might directly impact on intentions and changes in affective attitude might impact on the other attitudinal variables, and given the network model, a change in *Att1* provides a route to influence Pbc2, which should further strengthen the intentions. In essence the network reveals that for regular exercise behaviour among the student population, the affective attitudinal variable is the strongest node and therefore interventions could prioritise targeting changing the emotional responses to exercise to increase intentions to exercise. The network gives little support to intervening to change normative beliefs. This section indicates how network analysis in principle can influence not just how we appraise the pathways proposed in our theories, but also how it may offer guidance for interventions.

The present example aimed to highlight some of the key aspects to conducting network analysis in *R* and how to make sense of the outputs. Many real world networks estimated in psychology are likely to be messy and therefore interpretations require tempering in light of the stability and accuracy of the estimates. As network analysis becomes more prevalent, replication of network structures and properties will give greater confidence in the interpretations of the network patterns.

Of note, the psychosystems group has also developed an online web app (https://jolandakos.shinyapps.io/NetworkApp/) that allows researchers to visualise and analyze networks from data uploaded into the app. The app, based on the *R* packages describe above, can analyse data in different common formats (e.g. ‘.csv’, ‘.xls’ and ‘.sav’) and the data can represent the raw data, the correlation matrix between the variables, an adjacency matrix, or an edge list. The user can inform the app how missing data were coded and can also apply the non-paranormal transformation for data that are not normally distributed. The app provides the various options outlined in this paper for estimating the network structure from the raw data; these include the GLASSO, the graphical VAR, and multilevel VAR. The network default is to use the Fruchterman-Reingold Algorithm to layout the network and the user can decide various visual settings (e.g. size of nodes). It also calculates the centrality (strength, closeness and betweenness) indices to determine a node’s importance in the network. A clustering analysis can be run on the data and the networks from two groups can be compared. This resource offers a very user-friendly means to start to examine network structures in data.

## Conclusion

Barabási (2012) argued that theories cannot ignore the network effects caused by interconnectedness among variables. Health psychological processes reflect complex systems and to understand such systems, we need to understand the networks that define the interactions between the constituent variables. Many of our core health psychology models comprise networks of interacting constructs. Considering such psychological processes and outcomes from this perspective offers alternate ways of conceptualising and answering important psychological questions. Networks evolve over time due to dynamical processes that add or remove nodes (variables) or change edges (relationships between variables): the power of network science derives from the ability of the network to model systems where the nature of the nodes (e.g. symptoms, behaviours, beliefs, physiological arousal) and the edges (e.g. correlational relationship, causal relationship, social connection) can vary. Network analysis as a technique has been briefly outlined and how to conduct a simple analysis in *R* was presented. Hopefully this brief paper will encourage health psychologists to think about their data in terms of networks and to start to apply network analysis methods to their research questions. The work of Borsboom and colleagues provides a key foundation for network analyses and, as mentioned at the start of this paper, their invaluable contributions to the applications of network theory to psychology cannot be underestimated. Understanding the dynamic patterns of networks may offer unique insights into core psychological processes that impact health and well-being.

## Note

1 We wish to thank an anonymous reviewer for highlighting this possibility.
